# A small molecule inhibitor of XIAP induces apoptosis and synergises with vinorelbine and cisplatin in NSCLC

**DOI:** 10.1038/sj.bjc.6605418

**Published:** 2009-11-10

**Authors:** E J Dean, T Ward, C Pinilla, R Houghten, K Welsh, G Makin, M Ranson, C Dive

**Affiliations:** 1 Department of Clinical and Experimental Pharmacology, Paterson Institute for Cancer Research, University of Manchester, Wilmslow Road, Manchester M20 4BX, England, UK; 2 Derek Crowther Unit, Christie Hospital NHS Foundation Trust, Wilmslow Road, Manchester M20 4BX, England, UK; 3 Torrey Pines Institute for Molecular Studies, 3550 General Atomics Court, 2-129, San Diego, CA 92121-1122, USA; 4 Burnham Institute for Medical Research, La Jolla, CA 92037, USA

**Keywords:** XIAP antagonist compound, XIAP, XAC 1396-11, apoptosis, non-small cell lung cancer (NSCLC)

## Abstract

**Background:**

Evasion of apoptosis contributes to the pathogenesis of solid tumours including non-small cell lung cancer (NSCLC). Malignant cells resist apoptosis through over-expression of inhibitor of apoptosis proteins (IAPs), such as X-linked IAP (XIAP).

**Methods:**

A phenylurea-based small molecule inhibitor of XIAP, XIAP antagonist compound (XAC) 1396-11, was investigated preclincally to determine its ability to sensitise to clinically relevant cytotoxics, potentially allowing dose reduction while maintaining therapeutic efficacy.

**Results:**

XIAP protein expression was detected in six NSCLC cell lines examined. The cytotoxicity of XAC 1396-11 against cultured NSCLC cell lines *in vitro* was concentration- and time-dependent in both short-term and clonogenic assays. XAC 1396-11-induced apoptosis was confirmed by PARP cleavage and characteristic nuclear morphology. XAC 1396-11 synergised with vinorelbine±cisplatin in H460 and A549 NSCLC cells. The mechanism of synergy was enhanced apoptosis, shown by increased cleavage of caspase-3 and PARP and by the reversal of synergy by a pan-caspase inhibitor. Synergy between XAC 1396-11 and vinorelbine was augmented by optimising drug scheduling with superior effects when XAC 1396-11 was administered before vinorelbine.

**Conclusion:**

These preclinical data suggest that XIAP inhibition in combination with vinorelbine holds potential as a therapeutic strategy in NSCLC.

Lung cancer is the leading cause of cancer deaths among men and women. In the United Kingdom, lung cancer accounts for 22% of total cancer deaths with only a 7% 5-year survival (CR-UK, 2009). The major histological types of lung cancer are small cell lung cancer (SCLC), adenocarcinoma, squamous carcinoma and large cell carcinoma, the latter three of which are collectively referred to as non-small cell lung cancer (NSCLC). NSCLC represents approximately 80% of all cases and chemotherapy, most commonly consisting of a platinum agent in combination with another cytotoxic such as gemcitabine, vinorelbine or a taxane, can provide palliation and prolong survival (Spira and Ettinger, 2004). Despite encouraging progress with novel targeted agents, for example, erlotinib and bevacizumab, mortality rates remain dismal as over two-thirds of patients are diagnosed with advanced, metastatic disease for which no curative treatment is currently available.

One hallmark of cancer is evasion of apoptosis (Hanahan and Weinberg, 2000). Cancer cells are assumed to be under continuous pro-apoptotic stresses from genetic instability and their hostile microenvironment (oxygen and nutrient deprivation), but selected cell subpopulations adapt and de-couple these genetic and microenvironmental stress stimuli from commitment to apoptosis. The development of apoptosis-targeted therapies aims to lower the threshold for apoptosis but it is not yet clear whether these agents will be effective alone or in combination with chemotherapy or radiation (Reed, 2003).

One mechanism by which cells resist apoptosis is the over-expression of inhibitor of apoptosis protein (IAP) family of proteins. Although IAP proteins have shown diverse roles, it is their unique ability to inhibit distinct caspases that drive apoptotic cell death that has driven research exploring their therapeutic potential (Deveraux *et al*, 1997). Eight human IAPs have been identified of which X-linked IAP (XIAP) protein is the best characterised and most potent, inhibiting caspases-3 and -7 through its BIR2 domain (Scott *et al*, 2005) and caspase-9 through its BIR3 domain (Deveraux *et al*, 1997). To ensure cells commit to apoptosis when appropriately damaged, cells also use endogenous antagonists of XIAP (including SMAC, HtrA2/Omi, ARTS and XAF1) (Srinivasula *et al*, 2001b). These act by preventing XIAP binding to caspases (Du *et al*, 2000; Verhagen *et al*, 2000) or by triggering its redistribution from the cytosol to the nucleus (Liston *et al*, 2001). XIAP is considered to be a valid therapeutic target in malignancy because firstly it is over-expressed in the majority of a panel of NCI tumour cell lines (Tamm *et al*, 2000); secondly, XIAP over-expression correlates with resistance to apoptosis through stimulation of both the intrinsic (mitochondrial directed) and extrinsic (death receptor directed) pathways (Holcik *et al*, 2000; Wilkinson *et al*, 2004); thirdly, downregulation of XIAP with siRNA or antisense oligonucleotides (ASOs) restores chemosensitivity in various tumour cell lines (Sasaki *et al*, 2000; McManus *et al*, 2004); and finally, XIAP knockout mice have normal survival with no significant pathological features (Harlin *et al*, 2001), consistent with XIAP-targeted therapeutics exerting minimal toxicity to normal tissues.

XIAP inhibition, using small molecule inhibitors (SMIs) that target XIAP baculovirus (BIR) domains, gained momentum after the discovery of short polypeptides based on endogenous inhibitor SMAC, which are capable of selective BIR3 inhibition (Li *et al*, 2004; Oost *et al*, 2004). Subsequently, di- and tri-phenylurea-based XIAP antagonist compounds (XACs) that bind near the BIR2 domain have also been identified by combinatorial chemical library screening (Wang *et al*, 2004; Schimmer *et al*, 2004a). These XACs, but not inactive structural analogs, induced apoptosis directly in several haematologic and solid tumour cell lines *in vitro*, and sensitised cancer cells to chemotherapeutic drugs (Berezovskaya *et al*, 2005; Carter *et al*, 2005; Kater *et al*, 2005). Active compounds also suppressed the growth of established xenografts while displaying little toxicity (Karikari *et al*, 2007). Here, XAC 1396-11, a phenylurea-based SMI of XIAP (that targets near the BIR2 domain), was investigated as a potential therapy for NSCLC. The effect of XAC 1396-11 as a single agent and in combination with clinically relevant cytotoxic drugs was explored *in vitro*. The importance of optimising treatment schedule when inhibiting XIAP is shown, showing that treatment effects are, in part, dependent on when the ‘apoptotic brake’ is removed in relationship to a cytotoxic insult. These results suggest that XIAP inhibition with XAC 1396-11 holds promise as a therapeutic strategy in the treatment of NSCLC and that further studies are warranted.

## Materials and methods

### Human NSCLC cell lines

Human NSCLC cell lines H460, A549, H520, HCC827, H522 and HCT116 were obtained from the American Type Culture Collection (Manassas, VA, USA). MGH-4 and HCT116 XIAP−/− were kind gifts from Dr M-S Tsao (Princess Margaret Hospital, Toronto) and Dr B Vogelstein (Johns Hopkins University School of Medicine, MD, USA), respectively. All cells lines were maintained in RPMI 1640 supplemented with 10% fetal bovine serum.

### Reagents and chemicals

XAC 1396-11 was synthesised and purified as described (Schimmer *et al*, 2004b), and dissolved in DMSO for *in vitro* assays. All cytotoxics were purchased from Sigma-Aldrich (Gillingham, UK), except for gemcitabine (Eli Lilly, Basingstoke, UK). The pan-caspase inhibitor (Caspase Inhibitor III) was obtained from Calbiochem (Merck Chemicals Ltd, Nottingham, UK).

### Cell cytotoxicity

The sulforhodamine B (SRB) assay was used to determine cell population number in response to XAC 1396-11. NSCLC cell lines were plated in exponential growth phase in 96-well plates and treated with varying concentrations of XAC 1396-11. At various times, thereafter, cells were fixed and stained according to standard SRB protocol (Vichai and Kirtikara, 2006), and absorbance was measured using a microplate reader (Labsystems Multiskan EX, (Thermo Scientific, Milford, MA, USA) at 540 nm. Nuclear apoptotic morphology was assessed by UV-microscope examination of fixed cells stained with DAPI. Treated cells were trypsinised and re-suspended in PBS. The samples were cytospun onto slides at 500 r.p.m. for 5 min before fixing in 1% formaldehyde in PBS-T. The slides were washed in PBS-T and the cells stained with ProLong Gold antifade reagent with DAPI (Invitrogen, Paisley, UK). Slides were analysed by fluorescence microscopy (358/461 nm) using an Olympus BX51.

### Clonogenic assay

Cells were plated at 200 per well in six-well tissue culture plates (Costar, Corning, NY, USA) and allowed to attach overnight. Cells were treated with varying concentrations of XAC 1396-11 for 24 h, before the medium was aspirated, cells washed with PBS and fresh medium added. Plates were kept in a tissue culture incubator at 37°C and 5% CO_2_ for 7 days to allow colony growth. Colonies were fixed with 70% methanol and stained with methylene blue, and colonies (>50 cells) were counted. All assays were performed in triplicate. Surviving fraction was calculated as number of colonies in the test condition/number of colonies in the untreated well and plotted logarithmically against drug concentration.

### Immunoblot assay

For immunoblot analysis, cells were treated with XAC 1396-11 or with vehicle control for various times. Protein lysates were prepared using lysis buffer (10 × ) (Cell Signalling Technology, Danvers, MA, USA) and protease inhibitor cocktail (Sigma-Aldrich). All samples were sonicated at 10 Hz for 10 s. Protein lysates were resolved by electrophoresis in appropriate percentage polyacrylamide gels and transferred to PVDF membranes (Immobilon, Millipore, Watford, UK). Standard immunoblotting procedures were followed with overnight incubation at 4°C with the following primary antibodies: XIAP 1 : 1000 (BD Transduction Laboratories, Oxford, UK), cIAP-1 1 : 1000 (R&D Systems, Minneapolis, MN, USA), cIAP-2 (R&D Systems), Survivin (Novus Biologicals, Littleton, CO, USA), SMAC 1 : 1000 (BD Transduction Laboratories), XAF1 1 : 1000 (Imgenex, San Diego, CA, USA) and PARP (Cell Signalling). Blots were visualised with the enhanced chemiluminescence system (Amersham, Chalfont St Giles, UK) and analysed using a Fuji LAS-1000 Plus imaging system with AIDA software (Fuji, Bedford, UK). The proportion of cleaved caspase-3 was measured using the Meso Scale Discovery MULTI-SPOT Cleaved/Total Caspase-3 Assay.

### Drug combination assays

The combination index (CI) method was used to determine multiple drug–effect interaction using the computer software CalcuSyn (Biosoft, Cambridge, UK). The method is based on the multiple drug–effect equation of Chou–Talalay derived from enzyme kinetic models (Chou and Talalay, 1977) in which values for drug additivity are in the range CI=0.9–1.1 and values for synergy and antagonism are <0.9 and CI >1.1, respectively. The ratios of XAC 1396-11 and cytotoxic drugs were fixed using IC_50_ values from the SRB assay. Cells were co-treated for 72 h using XAC 1396-11 and various cytotoxic drugs. Six drug concentrations were used covering the concentration–effect. Linear correlation coefficients (*r*) were generated for each concentration response curve to determine the applicability of the data to the method of analysis. To confirm the synergistic drug interactions identified by CI analysis, the three-dimensional (3D) response surface model of Pritchard and Shipman (Pritchard and Shipman, 1990) was applied. The 3D method is based on a five by eight ‘checkerboard’ matrix (40 data points) of XAC 1396-11/cytotoxic drug combinations plus each drug tested alone, covering the concentration–effect curve. Data from the SRB assay, performed in triplicate, were used to calculate theoretical (individual dose responses) and experimental surfaces (drug combinations minus 2 s.d.). The theoretical surface is then subtracted from the experimental surface to reveal regions of synergy (above the baseline) and antagonism (below the baseline). Positive values are summed together to give the overall synergy value (*μ*M^2^%) across the response surface at the 95% confidence interval.

### Statistics

Statistical significance for a change in percentage cells with nuclear apoptotic morphology was determined by two-tailed paired *t*-tests (assuming equal variance) between the drug combination counts and the single agents.

## Results

### IAPs are widely expressed in NSCLC cell lines

**Figure 1:**
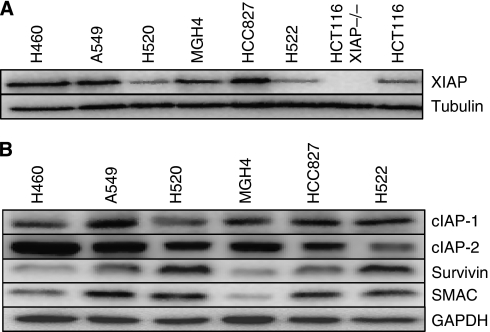
Immunoblot analysis of IAP family members in NSCLC lines. (**A**, **B**) Cell lysates were prepared from cell lines, normalised for total protein content (15 *μ*g per lane), and analysed by SDS–PAGE using antibodies recognising XIAP, c-IAP1, c-IAP2, Survivin and SMAC. Tubulin or GAPDH was used as a protein loading control. Data are representative of *n*=3 independent repeat experiments.

Immunoblot analysis of a panel of NSCLC cell lines confirmed that XIAP is widely expressed at levels comparable with the colon cancer cell lines HCT116, known to express XIAP at a level above the average reported for the NCI 60 tumour cell line panel (Figure 1) (Tamm *et al*, 2000). Additionally, cIAP-1, cIAP-2, Survivin and SMAC were also expressed in the NSCLC lines tested, albeit with levels varying among cell lines.

### XAC 1396-11 induces apoptotic cell death in a time- and concentration-dependent manner in NSCLC cell lines

**Figure 2:**
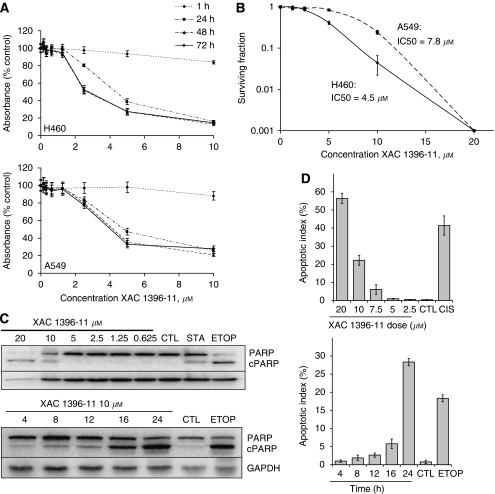
XAC 1396-11 induces cell death by apoptosis in NSCLC cells. (**A**) NSCLC cells were plated in exponential phase and treated at the indicated concentrations and times. Growth inhibition was determined by SRB assay (mean±s.e.m.; *n*=3). (**B**) Clonogenic survival determined after 24 h XAC 1396-11 at the indicated concentrations. Colonies were grown for 7 days before the plates were fixed. Surviving fraction was calculated as the number of colonies in the test condition divided by the number of colonies in the untreated well and plotted logarithmically against drug dose (mean±s.e.m.; *n*=3). (**C**) H460 cells were treated for 24 h at the indicated concentrations (upper panel) or at 10 *μ*M XAC 1396-11 for the times (h) shown (lower panel), before lysates were prepared for immunoblot analysis. Membranes were probed with antibodies specific for full-length or cleaved (‘c’ as indicated) PARP and for GAPDH. (**D**) H460 cells were treated as for (**C**), but fixed and DAPI stained for assessing percentage apoptotic nuclear morphology (mean±s.e.m.; *n*=3). CTL=control; ETOP=etoposide 20 *μ*M; STA=staurosporine 1 *μ*M; CIS=cisplatin 50 *μ*M.

XAC 1396-11 caused concentration-dependent growth inhibition in H460, A549, H520 and MGH-4 cell cultures (shown for H460 and A549 in Figure 2A), as measured by the SRB assay. The IC_50_ for a 72 h drug challenge was 2.8 and 4.0 *μ*M for H460 and A549 cells, respectively. In both of these cell lines, a 1 h drug exposure had minimal effect on growth inhibition but by 24 h, near maximal effect was observed. Similar growth inhibition was observed at 48- and 72 h exposure to XAC 1396-11.

The long-term effects of XAC 1396-11 were assessed by clonogenic assay (Figure 2B). The clonogenic IC_50_ values after 24 h XAC 1396-11 treatment were 4.5 and 7.8 *μ*M for H460 and A549 cells, respectively, showing higher sensitivity for H460 cells consistent with the SRB assay.

Analysis of the levels of cleaved PARP in XAC 1396-11-treated H460 cells confirmed time- and concentration-dependent activation of endogenous caspases, suggesting that the observed cell loss occurred by apoptosis (Figure 2C). To provide further confirmatory evidence of apoptotic cell fate, H460 cells treated in both a time- and concentration-dependent manner were also stained with DAPI to reveal nuclear morphology. Concentrations of XAC 1396-11 ⩾7.5 *μ*M were sufficient to induce apoptotic morphology after 24 h drug exposure (Figure 2D upper panel). Increased apoptotic indices were evident as early as 16 h after treatment with 10 *μ*M XAC 1396-11 (Figure 2D, lower panel).

### XAC 1396-11 synergises with cytotoxic anticancer drugs in NSCLC lines

**Figure 3:**
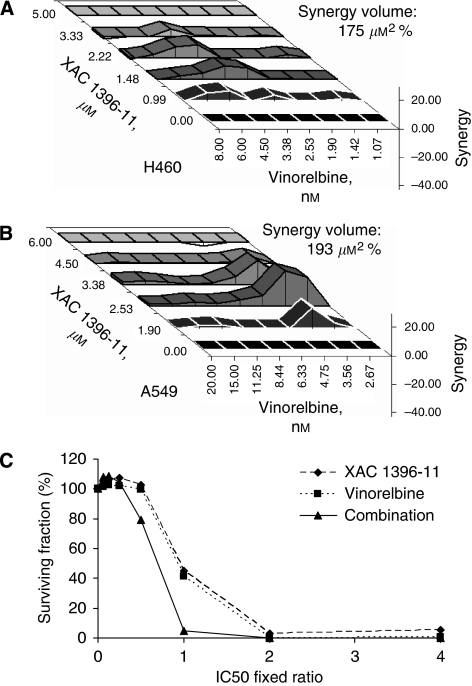
XAC 1396-11 synergises with cytotoxic anticancer drugs in H460 and A549 cell lines. (**A**) H460 (**A**) and A549 (**B**) cells were treated in a five by eight checkerboard matrix of XAC 1396-11/cytotoxic combinations, plus each drug tested alone, covering the concentration–effect curve. After 72 h, plates were fixed and analysed by SRB before determining synergy using the three-dimensional Pritchard and Shipman model. Positive values (above the baseline) represent synergy; negative values (below the surface) are antagonistic. The overall synergy value (*μ*M^2^%) is shown at the 95% confidence interval. (**C**) H460 cells were treated as in Table 1. The *x* axis shows the fixed ratio used in the combination with equipotent concentrations of the single agents. The combination arm shifts the concentration–effect curve to the left. All experiments were repeated in triplicate, error bars show s.e.m.

Pre-clinical data on apoptosis-targeted agents suggest that their ultimate clinical use will be in combination with cytotoxics, in which an enforced reduction in the cellular threshold for apoptosis by the former should sensitise to the latter. Therefore, the effects of XAC 1396-11 in combination with a variety of chemotherapeutics that are conventionally used in NSCLC were explored using the CI and Pritchard and Shipman methods. Table 1 shows the CI values derived from treatment with XAC 1396-11 and various cytotoxic anticancer drugs. XAC 1396-11 showed synergy with vinorelbine, cisplatin and gemcitabine in both H460 and A549 cell lines. However, the combination of XAC 1396-11 with taxotere was not synergistic. Particularly, striking was the synergy at IC_25_, IC_50_ and IC_75_ observed for the combination of XAC 1396-11 and vinorelbine in both the H460 and A549 cell lines after 72 h co-administration. Synergy was also noted in the concentration–effect graph (Figure 3C) as a left shift for the combination treatment, that is the surviving fraction is less for the combination using the same concentrations compared with single drug treatments. In H460 cells, the XAC 1396-11/vinorelbine combination was also synergistic at 24 and 48 h drug exposure (data not shown). Further confirmatory evidence of synergy between XAC 1396-11 and vinorelbine was shown using the Pritchard and Shipman method. Figures 3A and B show the 3D checkerboard plots from combining 5 dilutions of XAC 1396-11 (dilution factor 1.5 in H460 cells, 1.3 in A549) with 8 dilutions of vinorelbine (dilution factor 1.3 in both cell lines). The most synergistic concentrations were 2.2 *μ*M XAC 1396-11 and 3.4 nM vinorelbine in H460 cells, and 2.5 *μ*M XAC 1396-11 and 3.6 nM vinorelbine in A549 cells. The overall synergy values show that over the entire response surface, synergy was comparable between the two cell lines, H460 175 *μ*M^2^% and A549 cells 193 *μ*M^2^% at the 95% confidence interval.

### XAC 1396-11 and vinorelbine induce schedule-dependent apoptotic cell death of H460 cells

**Figure 4:**
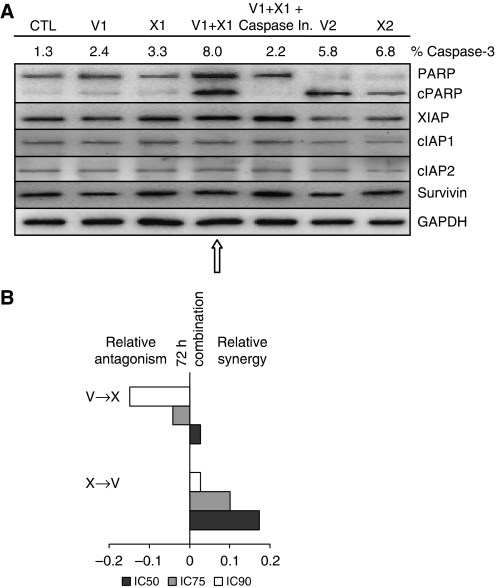
Combination of XAC 1396-11 and vinorelbine synergistically induces apoptosis of NSCLC cells. (**A**) H460 cells were treated for 72 h with vinorelbine, XAC 1396-11 or the combination±pan-caspase inhibitor (caspase In.), at the indicated concentrations below. Lysates were then prepared for immunoblot analysis using antibodies recognising PARP (full-length and cleaved ‘c’ PARP), XIAP, cIAP1, cIAP2, Survivin and GAPDH. Low concentrations of the single agents failed to induce PARP cleavage, whereas combination treatment provoked PARP cleavage (arrow). V1=vinorelbine 2.5 nM; X1=XAC 1396-11 5 *μ*M; Caspase In.=50 *μ*M; V2=vinorelbine 10 nM; X2=XAC 1396-11 10 *μ*M; CTL=control (**B**). H460 cells were treated for 36 h with the first agent followed by removal of the supernatant and treatment for 36 h with the second agent, covering the concentration–effect curve (V=vinorelbine; X=XAC 1396-11). The plates were fixed and growth inhibition was determined by SRB. The CI results at the IC_25_, IC_50_ and IC_75_ are shown. All experiments were repeated in triplicate, error bars show s.e.m.

To determine whether the observed synergy between XAC 1396-11 and vinorelbine resulted from enhanced apoptosis, H460 cells were exposed to either of the single agents at low concentrations that did not result in a significant elevation in cleaved caspase-3 or PARP. The combination of XAC 1396-11 and vinorelbine, at the same low concentrations, increased cleaved caspase-3 and induced PARP cleavage (Figure 4A arrow). Significantly, higher apoptotic index values were seen for the combination than either single agent alone (*P*<0.01), data not shown. This process could be reversed by the addition of a pan-caspase inhibitor providing further evidence that synergy is mediated through apoptosis. Immunoblot analysis shows that in cells treated with low concentrations of XAC 1396-11, no compensatory increases the levels of XIAP, c-IAP1, c-IAP2 and Survivin were observed (confirmed by densitometry measurements).

### Schedule dependency of vinorelbine and XAC 1396-11 synergy

To determine whether the sequence of drug addition impacts on resulting synergy, cells were treated for 36 h with XAC 1396-11, followed by removal of the supernatant, and treatment for 36 h with the vinorelbine, or vice versa. The CI results show that treatment with XAC 1396-11 followed by vinorelbine is more synergistic than sequencing these agents in the reverse order, or for 72 h co-treatment, (Figure 4B).

### XAC 1396-11, cisplatin and vinorelbine are a synergistic drug regimen

As cisplatin and vinorelbine are conventionally used together in the treatment of NSCLC, the impact of both cytotoxics with XAC 1396-11 was investigated (Table 1). The three drug combination showed greatest synergy at low concentrations IC_25_ consistent with the hypothesis that XAC 1396-11 can sensitise to clinically relevant cytotoxics, potentially permitting dose-reduction while maintaining efficacy.

## Discussion

NSCLC is the most prevalent cancer worldwide yet mortality rates remain dismal. Although some progress has been made in the development of targeted agents for NSCLC, palliative chemotherapy remains the mainstay of treatment for the majority of patients who present with advanced metastatic disease and who often have co-morbidities making them unfit for radical surgery or chemo-radiation. After a large-scale combinatorial screen of chemical libraries, Schimmer *et al* (2004a) discovered multiple small molecules with XIAP inhibitory activity, including the compound XAC 1396-11. The preclinical effects of these XIAP inhibitors have been confirmed by their ability to induce apoptosis of both haematologic and solid tumour cell lines *in vitro*, and their ability to sensitise cancer cells to chemotherapeutic drugs (Schimmer *et al*, 2004a; Carter *et al*, 2005). Our rationale was to investigate whether XIAP inhibition with XAC 1396-11, in NSCLC, could sensitise to chemotherapy with the potential to dose-reduce cytotoxic treatment, thus preventing chemotherapy-induced toxicity but maintaining therapeutic efficacy.

This is the first report to explore the efficacy of a small molecule XIAP antagonist in NSCLC. Using XAC 1396-11 in combination with cytotoxics *in vitro*, evidence is presented that XIAP inhibition sensitises cancer cells to death induced by a variety of chemotherapeutic agents. This cytotoxic effect was most pronounced with vinorelbine in H460 and A549 NSCLC lines. Furthermore, the synergy data for the XAC 1396-11/vinorelbine combination are consistent using two differing methodologies in which the mechanism of synergy is through enhanced apoptosis, as shown by cleavage of caspase-3 and PARP and by the reversal of synergy using a pan-caspase inhibitor. Vinorelbine, a *Vinca* alkaloid, interferes with microtubule assembly leading to mitotic arrest and/or cell death. It is approved for use as a single agent or in combination with platinum in the first line treatment of stage III or IV NSCLC. Our results suggest the addition of XAC 1396-11 to vinorelbine alone or in combination with a platinum agent may sensitise patients to the effects of chemotherapy. The drug sequencing experiments reported here data support the notion that vinorelbine is most effective at inducing apoptosis once the ‘apoptotic brake’ has been removed with XAC 1396-11. The data presented show that at every inhibitory concentration, that is IC_25_, IC_50_ and IC_75_, pretreatment with XAC 1396-11 followed by vinorelbine was more synergistic than the reverse sequence. This supports the concept that blockade of an endogenous apoptosis suppressor allows drug damage to be coupled to the engagement of apoptosis.

XAC 1396-11 targets near the BIR2 domain of XIAP (Schimmer *et al*, 2004a), which is responsible for inhibition of caspases-3 and -7, down-stream effector proteases operating at the convergence of both intrinsic and extrinsic apoptotic pathways. However, it is not clear whether blocking BIR2 to mediate release of effector caspases, or selectively targeting the BIR3 domain, which binds to inhibit the upstream initiator protease caspase-9, is the more effective strategy (Srinivasula *et al*, 2001a; Nikolovska-Coleska *et al*, 2004; Sun *et al*, 2006). It has been shown in H460 cells that disruption of XIAP–caspase-9 binding with SMAC mimetics restored apoptosis (Yang *et al*, 2003). However, intratumoural injections of SMAC mimetics alone in an H460 xenograft model did not have any apparent tumour-suppressive effect. Theoretically, SMIs that target both the BIR2 and BIR3 domains should be most efficacious. In interpreting the results of experiments with IAP-family proteins, it should be noted that complex effects have been documented beyond reversal of caspase inhibition. For example, compounds that target SMAC-binding sites on BIR domains activate the intrinsic E3 ligase activity of IAPs, causing their self-ubiquitinylation and subsequent proteasome-dependent degradation (Zhang *et al*, 2004). Compound-triggered clearance of cIAP1 and cIAP2 from cells causes accumulation of NIK and induces other events that stimulate NF-*κ*B activity and induce TNF*α* production (Varfolomeev *et al*, 2007). Recently, it has also been shown that efficient induction of cell death by SMIs requires antagonism of both c-IAPs and XIAP proteins but whether this pan-IAP inhibition is associated with higher toxicities remains untested (Ndubaku *et al*, 2009). Further analysis of the phenylurea-series XIAP inhibitors, such as XAC 1396-11, is required to ascertain their effects on caspase-independent aspects of XIAP biology (such as NF-*κ*B activation mediated by TAK/TAB binding) and to elucidate whether they target other members of the IAP or have off-target activities that contribute to their cytotoxic activity.

Another approach to targeting XIAP is the use of small interfering RNA (siRNA) to induce degradation of the target mRNA. XIAP siRNA enhances sensitivity to methotrexate in hepatoma (Chen *et al*, 2006), to cisplatin, fluorouracil and etoposide in oesophageal carcinoma (Zhang *et al*, 2007), to TRAIL in melanoma, breast cancer and pancreatic cancer (Chawla-Sarkar *et al*, 2004; McManus *et al*, 2004) and also sensitises pancreatic cancer cell lines to γ-irradiation (Giagkousiklidis *et al*, 2007). The XIAP siRNA approach confirmed the validity of XIAP as a therapeutic target but the problem of *in vivo* delivery of siRNAs regimens still must be overcome.

An alternative nucleic acid-directed strategy for targeting XIAP is the use of ASOs that form a duplex with native mRNA, inducing its degradation through RNAase H enzymes. The undertaking of this study was encouraged by reports that XIAP downregulation in lung cancer using ASOs can be synergistic with other therapeutic modalities used in the treatment of the disease. XIAP ASOs have also been combined with cytotoxics (doxorubicin, paclitaxel, vinorelbine and etoposide) in H460 cells *in vitro*, showing synergy using the CI method. In H460 tumour-bearing mice, XIAP ASOs have also been combined with vinorelbine (Hu *et al*, 2003) and *γ*-irradiation (Cao *et al*, 2004), showing a significant delay in tumour establishment and reduction in tumour volume, respectively. In addition, *in vivo* tumour xenograft models of prostate (PC-3), colon (LS174T) and NSCLC (H460) cancer showed that XIAP ASO was effective as a single agent (LaCasse *et al*, 2006). Phase I/II trial results for XIAP ASOs both as a single agent and in combination with chemotherapy in refractory/relapsed AML have recently been published using a variety of dosing schedules (AEG35156, Aegera Therapeutics Inc. Montreal, Québec, Canada) (Dean *et al*, 2009; Schimmer *et al*, 2009). AEG35156 was well tolerated in the 7-day and 3-day infusion regimens with predictable toxicities (raised hepatic enzymes, hypophosphatemia and thrombocytopenia), pharmacokinetic properties and clinical evidence of antitumour activity. These early clinical data bode well for SMIs targeting XIAP that are approaching the clinic.

In summary, the results presented here are the first to address the potential therapeutic role of XAC 1396-11, an SMI of XIAP for NSCLC, and the possibility for synergy with cytotoxics commonly used in the treatment of the disease. Using two independent methods, XAC 1396-11 was shown to synergise with clinically used cytotoxic agents in NSCLC cell lines and evidence was obtained that the mechanism of synergy is through increased apoptosis. Pretreatment with the XIAP-targeting drug produced optimal synergy. Thus, combining small molecule therapeutics targeting apoptosis regulators, such as XIAP with conventional cytotoxic agents, promises to improve the management of highly resistant malignancies such as lung cancer, warranting further pre-clinical *in vivo* studies to evaluate the potential of such combination therapies.

## Figures and Tables

**Table 1:** XAC 1396-11 synergizes with cytotoxic anticancer drugs in H460 and A549 cell lines

**Cell line**	**XAC 1396-11 Combination**	**IC_25_**	**IC_50_**	**IC_75_**
H460	Vinorelbine	0.40 (+++)	0.37 (+++)	0.35 (+++)
H460	Cisplatin	0.80 (++)	0.74 (++)	0.50 (+++)
H460	Vinorelbine and cisplatin	0.65 (+++)	0.70 (++)	0.74 (++)
H460	Gemcitabine	0.72 (++)	0.74 (++)	0.87 (+)
H460	Taxotere	1.02 (±)	1.09 (±)	1.11 (−)
A549	Vinorelbine	0.36 (+++)	0.24 (++++)	0.33 (+++)
A549	Cisplatin	0.84 (++)	0.79 (++)	0.73 (++)
A549	Vinorelbine and cisplatin	0.69 (+++)	0.78 (++)	0.85 (+)
A549	Gemcitabine	0.78 (++)	0.93 (±)	1.09 (±)
A549	Taxotere	1.08 (±)	1.04 (±)	1.02 (±)

Non-small cell lung cancer (NSCLC) cells were treated for 72 h with equipotent concentrations of XAC 1396-11 and the cytotoxic, before being fixed and analysed by SRB assay. Six concentrations of drugs were used starting at 4 × IC_50_ values and covering the dose–effect curve. Additivity is taken to be in the range CI=0.9–1.1 (±), synergy CI <0.9 (+) and antagonism CI >1.1(−).
